# Estimation of the Share of Net Expenditures on Insulin Captured by US Manufacturers, Wholesalers, Pharmacy Benefit Managers, Pharmacies, and Health Plans From 2014 to 2018

**DOI:** 10.1001/jamahealthforum.2021.3409

**Published:** 2021-11-05

**Authors:** Karen Van Nuys, Rocio Ribero, Martha Ryan, Neeraj Sood

**Affiliations:** 1 Leonard D. Schaeffer Center for Health Policy & Economics, Price School of Public Policy, University of Southern California, Los Angeles

## Abstract

**Importance:**

Recent US media and policy attention on insulin affordability has focused on the role of manufacturers in setting prices; however, the role of other drug distribution intermediaries in determining prices has received less attention.

**Objective:**

To estimate the share of net expenditures on insulin captured by manufacturers, wholesalers, pharmacy benefit managers, pharmacies, and health plans from 2014 to 2018.

**Design, Setting, and Participants:**

This cross-sectional study of the US insulin market was conducted in 2020 using 2014-2018 data from multiple sources, including list and estimated net prices from SSR Health for 32 insulin products, mean use weights from a commercial pharmacy claims database, mean acquisition costs and reimbursements from the Centers for Medicare & Medicaid Services, mean spread and share of rebates retained by pharmacy benefit managers from state Medicaid and drug transparency reports, and profit margins from the public filings of distribution system participant companies. Participants were insulin manufacturers, drug wholesalers, pharmacies, pharmacy benefit managers, and health plans.

**Main Outcomes and Measures:**

Mean list and net insulin prices, mean net expenditures on insulin, share of expenditures retained by each distribution system participant.

**Results:**

Between 2014 and 2018, mean list prices of 32 insulin products increased by 40.1% (from $19.60 to $27.45), while mean net prices received by manufacturers decreased by 30.8% (from $10.53 to $7.29). Net expenditures per 100 units of insulin increased by 3.2% (from $15.11 to $15.59) while the share of a hypothetical $100 insulin expenditure accruing to manufacturers decreased by 33.0% (from $69.71 to $46.73) and the share accruing to health plans decreased by 24.7% (from $13.82 to $10.40). The share of insulin expenditures retained by pharmacy benefit managers increased by 154.6% (from $5.64 to $14.36), the share retained by pharmacies increased by 228.8% (from $6.21 to $20.42), and the share retained by wholesalers increased by 74.7% (from $4.63 to $8.09).

**Conclusions and Relevance:**

Results of this cross-sectional study of the distribution of insulin expenditures suggest that policies to control insulin costs should consider all entities throughout the insulin distribution system. Manufacturers represented a decreasing share of insulin expenditures, and pharmacies, pharmacy benefit managers, and wholesalers accounted for a growing share; all entities play a role in increasing insulin costs.

## Key Points

**Question:**

How are net expenditures on insulin distributed across manufacturers, wholesalers, pharmacies, pharmacy benefit managers, and health plans in the pharmaceutical distribution system?

**Findings:**

In this cross-sectional study of the distribution of insulin expenditures between 2014 and 2018, the list price of insulin increased, and the net price received by manufacturers decreased. The share of insulin expenditures flowing to manufacturers decreased, and the share flowing to distribution system intermediaries increased, particularly among pharmacies and pharmacy benefit managers.

**Meaning:**

Results of this cross-sectional study suggest that commercial practices of all distribution system participants influence insulin costs, and that policies to control insulin costs should consider all participants, including pharmacy benefit managers and pharmacies, who share responsibility for rising insulin costs.

## Teaser

This cross-sectional study uses data sources to estimate the net expenditures on insulin across the pharmaceutical distribution system and which entities capture what share of those expenditures, through their involvement in insulin distribution.

## Introduction

Increasing insulin prices have drawn recent media and government scrutiny.^1,2^ List prices of insulin products doubled between 2012 and 2016 and continue to increase,^3,4,5,6^ causing patients to face large out-of-pocket (OOP) costs.^7,8^ Patients who cannot afford these OOP costs may resort to rationing insulin, which has been linked to poor health outcomes and death.^8,9,10^ Lawmakers are seeking explanations: Senators Grassley and Wyden launched a bipartisan investigation in 2019, issuing their final report in 2021,^2,11^ and House and Senate committees have held hearings on high insulin prices.^12,13^ These policy makers have focused on patient and taxpayer financial stress due to rising prices, and the mystery of sharply increasing prices for a drug developed almost 100 years ago.

Beyond increasing list prices (the undiscounted price set by the manufacturer), several analyses found a widening gap between list and net prices (what the manufacturer receives after accounting for all discounts and payments to distribution system entities).^3,11,14^ Some insulin product list prices have been increasing as their net prices have been decreasing.^11,15,16,17^ This divergence between list and net prices suggests that intermediaries such as wholesalers, pharmacy benefit managers (PBMs), and retailers are earning higher rebates and discounts from manufacturers. Thus, although manufacturers set list prices, intermediaries might be the primary beneficiaries of their increase.^18^

The share of total drug expenditures captured by different distribution system players has been documented for retail drugs overall, with roughly equal shares accruing to manufacturers and to intermediaries taken together.^19^ However, trade secret and other protections imposed by distribution system participants have prevented outsiders from knowing who profits from the distribution of particular products, including insulin, and the total costs being paid through third-party payers. To better understand the opaque insulin distribution system, the American Diabetes Association convened the Insulin Access and Affordability Working Group in 2017, which recommended increasing transparency as a first step to improve insulin affordability.^3^

Although comprehensive legislation providing transparency at each stage of the drug distribution system has not been passed at the national or state level,^20^ some state governments have passed initiatives to gather selected information. Recently compiled data from state drug transparency initiatives in Nevada and Ohio offer greater insight into the economics of the diabetes drug distribution system.^21,22,23^ We combine these data with insulin list and net price data from a third-party vendor^24^ and data on profit margins from financial statements of public distribution system companies to estimate the flow of funds through the insulin distribution system using previously published methods.^19^ By piecing together parameters from these and other sources, we can estimate the net expenditures on insulin and which entities capture what share of those expenditures through their involvement in insulin distribution.

## Methods

### Study Design

For this cross-sectional study, we used an approach similar to that in Sood et al^19^ to estimate the flow of funds through the insulin distribution system using, wherever possible, data specific to insulin products. In some cases in which insulin-specific values were not available, we used parameters specific to diabetes drugs.

**Figure 1.**
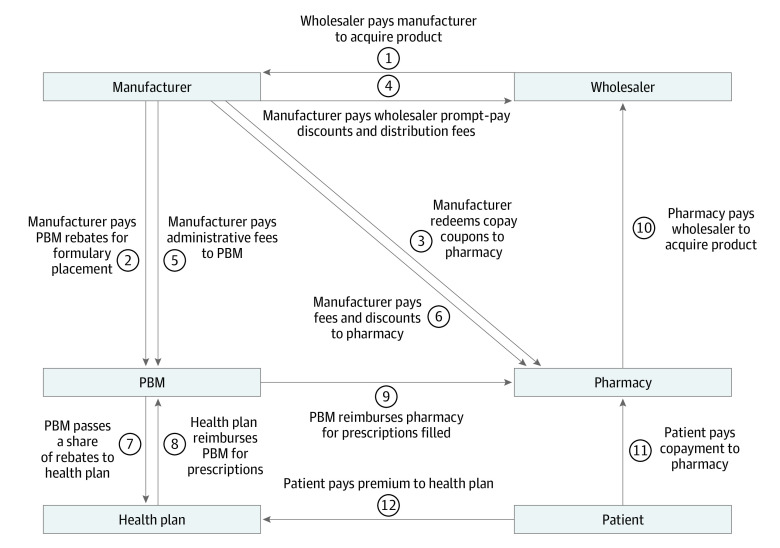
Conceptual Diagram of Money Flows in the Pharmaceutical Distribution System Abbreviation: PBM, pharmacy benefit manager. Adapted from Sood N, et al^19^ with permission from USC Leonard D. Schaeffer Center for Health Policy & Economics.

Figure 1 illustrates a stylized representation of money flows among insulin distribution system participants, characterized as flows 1 through 12. Each flow represents a payment or discount from one party to another, and we estimated the mean magnitude of each flow for the insulin products studied using sources and methods described in the Table and eMethods and eTable 2 in the Supplement. Product-specific flows were weighted by the share of insulin claims in each year represented by each product to calculate a weighted mean value across 32 insulin products.

**Table.** Sources and Methods Used to Estimate Money Flows in the Pharmaceutical Distribution System

Flow	Sources	Methods/notes
1. Wholesaler pays manufacturer to acquire product	SSR Health	Mean wholesale acquisition costs for 32 insulin products, weighted by use in commercial claims.
2. Manufacturer pays rebate to PBM for formulary placement	SSR Health, DCI Reports, MACPAC Medicaid Drug Spending Reports, CMS Rebate Summary for Brand Name Drugs	Rebates as a share of the gross-to-net reduction (calculated from SSR data) taken from DCI reports in 2017 and 2019, extrapolated in other years. The eMethods and eTable 3 in the Supplement include details.
3. Manufacturer redeems copay coupons to pharmacy	SSR Health, DCI Reports, MACPAC Medicaid Drug Spending Reports, CMS Rebate Summary for Brand Name Drugs	Manufacturer payments to copay offset programs taken from DCI reports; copay offset payments as a share of total manufacturer payments to intermediaries calculated. The eMethods and eTable 3 in the Supplement include details.
4. Manufacturer pays wholesaler prompt-pay discounts and distribution fees	SSR Health, DCI Reports, MACPAC Medicaid Drug Spending Reports, CMS Rebate Summary for Brand Name Drugs	60% (48%, 51%, 50%, 50%) of the remaining gross-to-net reduction after rebates and copay offset payments go to wholesalers in 2014 (2015, 2016, 2017, 2018, respectively), based on their reported gross margins. The eMethods and eTable 3 and eTable 4 in the Supplement include details.
5. Manufacturer pays administrative fees to PBM	SSR Health, DCI Reports, MACPAC Medicaid Drug Spending Reports, CMS Rebate Summary for Brand Name Drugs	20% (26%, 24%, 25%, 25%) of the remaining gross-to-net reduction after rebates and copay offset payments go to PBM administrative fees in 2014 (2015, 2016, 2017, 2018, respectively). The eMethods and eTable 3 and eTable 4 in the Supplement include details.
6. Manufacturer pays fees and discounts to pharmacy	SSR Health, DCI Reports, MACPAC Medicaid Drug Spending Reports, CMS Rebate Summary for Brand Name Drugs	20% (26%, 24%, 25%, 25%) of the remaining gross-to-net reduction after rebates and copay offset payments go to pharmacy fees and discounts in 2014 (2015, 2016, 2017, 2018, respectively). The eMethods and eTable 3 and eTable 4 in the Supplement include details.
7. PBM passes share of manufacturer rebates to health plan	Nevada Transparency Reports	PBMs retained 4.59% of essential diabetes drug rebates in 2017 and 6.59% in 2018 (assume earlier years were the same as 2017). Assume nonretained rebates are passed to insurers. Rebates estimated as in flow 2.
8. Health plan reimburses PBM for prescriptions filled	Medicare Part D Claims 2014-2018	Extracted weighted mean total cost minus OOP from Medicare Part D claims.
9. PBM reimburses pharmacy for prescriptions filled	Ohio Auditor of State report on Ohio’s Medicaid Managed Care Program	On average, 0.8% of payments made by plans for brand-name drugs were kept by PBMs as spread; remaining 99.2% of payments from plans was used to reimburse pharmacies.
10. Pharmacy pays wholesaler to acquire product	CMS–NADAC files	National average drug acquisition costs for 32 insulin products, weighted by use in commercial claims.
11. Patient pays copayment to pharmacy	Medicare Part D Claims 2014-2018	Estimated mean patient OOP for 32 insulin products from 2014-2018 claims data.
12. Patient pays premium to health plan	All	Insurance premiums are calculated as the net expenditures on the drug from all sources less the estimated patient OOP.

Abbreviations: CMS, Centers for Medicare & Medicaid Services; DCI, Drug Channels Institute; MACPAC, Medicaid and CHIP Payment and Access Commission; NADAC, National Average Drug Acquisition Cost; OOP, out of pocket; PBM, pharmacy benefit manager.

Of particular interest is the difference between the gross price manufacturers receive from wholesalers (flow 1) and the net price they keep after paying all fees and discounts to distribution system intermediaries, sometimes referred to as the gross-to-net bubble.^25^ We calculated this difference for each insulin product as the difference between list price and estimated net price, using SSR Health data, per 100 units of insulin. Prices of products containing 200, 300, or 500 units of insulin per 1 mL of solution were normalized by dividing them by 2, 3, or 5, respectively, so that normalized prices represent the cost per 100 units of insulin. We decomposed this gross-to-net reduction into its constituent parts, represented by the flows emanating from the manufacturer as rebates, payments, and discounts to PBMs (flows 2 and 5), pharmacies (flows 3 and 6), and wholesalers (flow 4). For this decomposition we used data from Drug Channels Institute (DCI) reports and Medicaid drug spending trends reports from the Medicaid and CHIP Payment and Access Commission. Using data from DCI and others, we computed the share of each year’s total gross-to-net reduction that went to patient assistance and copayment support, Medicaid rebates, Medicare Part D rebates, commercial payer rebates, and other fees and discounts, and applied these shares to the gross-to-net reduction for each insulin product, resulting in estimated magnitudes for flows 2 through 6. The eMethods in the Supplement report further details.

Values for flows 1 and 7 through 12 in Figure 1 were estimated using insulin-specific or diabetes drug–specific data wherever possible. Transparency reports published by the Nevada Department of Health and Human Services found PBMs retaining 4.59% of rebates on essential diabetes drugs in 2017 and 6.59% in 2018.^21,22^ We assumed that the 4.59% value also applied to 2014-2016, and that all nonretained rebates were passed to health plans (flow 7). Rebates paid by manufacturers to PBMs (flow 2) were calculated as described in the eMethods in the Supplement. To estimate health plan reimbursements for insulin prescriptions (flow 8), we used Medicare Part D non–low-income-subsidy claims to calculate the difference between total costs and patient OOP payments. To estimate the amount that PBMs reimbursed pharmacies for insulin prescriptions (flow 9), we used the finding of the Ohio Auditor of State that, on average, PBMs in managed Medicaid programs kept 0.8% of payments from health plans for brand-name drugs as spread and used the other 99.2% to reimburse pharmacies.^23^ We used the National Average Drug Acquisition Cost (NADAC) for each insulin product, weighted by use from commercial claims data, to estimate the amount paid by pharmacies to acquire insulin products from wholesalers (flow 10).

In addition, we calculated mean OOP payments (flow 11) for 32 insulin products from Medicare Part D claims from 2014-2018. Because patient OOP and premium payments comprise the only funds flowing into the system, and these inflows financed all the other flows, we calculated health plan premiums (flow 12) as the difference between the net expenditures on the drug and the patient OOP.

We compared inflows to and outflows from each entity in Figure 1 to estimate the dollar amount each entity kept from each 100 units of insulin sold, after all discounts and rebates. We defined net expenditures for insulins as the sum of all amounts retained by all distribution system entities. That is, we modeled a closed system, in that all resources put into it from external sources (patients and their insurance plan sponsors, including the government, which may subsidize premiums) were fully distributed across all system participants.

The analysis was conducted in the fall of 2020 and our report follows the Strengthening the Reporting of Observational Studies in Epidemiology (STROBE) reporting guideline for cross-sectional studies.^26^ The University of Southern California Institutional Review Board determined that the study met the criteria for coded private information or biological specimens and thus was exempt from informed consent requirements. All analyses were performed using Stata, version 14.0 (StataCorp LP).

### Data Analysis

The analysis relied on several recently published data sources for parameter values. We used parameter estimates specific to insulin whenever available; when they were not, we used estimates based on all diabetes drugs, or, lacking those, on all brand-name drugs.

#### SSR Health

The SSR Health Rx Brand Pricing Data Tool^24^ provided quarterly US list prices and estimated US net prices for brand-name drugs representing $380 billion in 2016 sales (about 84% of US brand-name prescription drug sales). SSR Health compiles information including product-level net revenues reported by publicly traded pharmaceutical firms in financial statements and earnings calls, and product-level unit volumes and list prices from Symphony Health. From SSR data, we identified all insulin products with strengths of 100, 200, 300 or 500 units per 1 mL of solution in vial or pen form with pricing data at any point during 2014 to 2018 (32 products corresponding to 43 National Drug Codes [NDCs]) (eTable 1 in the Supplement). After cross-referencing these NDCs with commercial pharmacy claims data from Optum Clinformatics Data Mart from 2007 to 2019, we excluded those with fewer than 100 claims.

We created annual mean wholesale acquisition costs (WAC, or list price) and net prices for each insulin product (defined by its brand name) from the SSR quarterly estimates and calculated for each product the difference between list and estimated net prices from 2014 to 2018. Using data from the Centers for Medicare & Medicaid Services (CMS), state drug legislation, public filings, and DCI, as well as sources listed in the eMethods and eTable 2 in the Supplement, we estimated the allocation of these product-specific differences between gross and net prices across distribution system participants.

#### Centers for Medicare & Medicaid Services

We collected the NADAC per milliliter published by CMS for included NDCs and calculated weighted annual mean acquisition costs for each insulin product, weighting each NDC by the number of commercial claims in each year. We analyzed Medicare Part D non–low-income-subsidy claims from 2014-2018 for the 43 NDCs to retrieve the mean total cost and patient OOP expenditures (defined as the sum of “patient payment amount” and “other true out-of-pocket”) for each insulin product.

#### State Drug Transparency Initiatives

In 2018, the Ohio Auditor of State published a report on its audit of payments made to PBMs for providing pharmacy services to the Ohio Medicaid managed care program.^23^ The report revealed that PBMs were keeping, on average, 0.8% of the payments made by the plans to the PBM for brand-name drugs, in the form of spread payments, with the other 99.2% used to reimburse pharmacies for filling prescriptions of Ohio Medicaid beneficiaries.

In 2017, Nevada passed legislation calling for PBMs and manufacturers to provide confidential data on prices, costs, rebates, and other financial variables for essential diabetes medicines, including insulin. State reports from 2017 and 2019^21,22^ revealed that PBMs retained, on average, 4.59% and 6.59% of the rebates collected from manufacturers for essential diabetes medicines in 2017 and 2018, respectively. The remaining rebates were passed through to insurers.^21,22^

#### Public Filings

We collected financial information from the 2014-2018 10-K and 20-F Securities and Exchange Commission filings of the 10 largest publicly traded health insurers and the 3 largest drug wholesalers. Replicating the analysis in an earlier study,^19^ we estimated gross margins as the difference between revenues and cost of goods sold as a percent of revenues. Health insurers do not report pharmaceutical and medical costs separately; our estimates of insurer margins therefore included all health expenditures, and our estimates of wholesaler margins were for all drugs.

#### Drug Channels Institute

In addition, we used estimates from DCI to calculate ratios to decompose gross-to-net reductions into their constituent parts. The DCI reports compile data from multiple sources, including CMS, US Government Accountability Office, SSR, and IQVIA to identify trends in pharmaceutical distribution.

## Results

### List Price, Net Price, and Net Expenditures for Insulin

**Figure 2.**
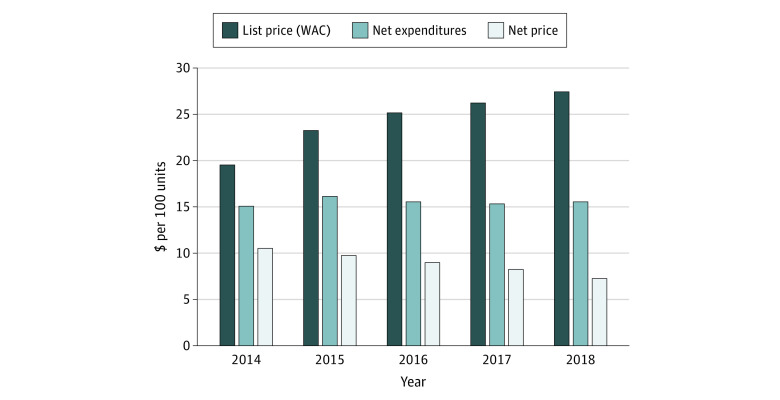
Mean List Price, Net Price, and Net Expenditures on 32 Insulin Products, 2014-2018 Abbreviations: CMS, Centers for Medicare & Medicaid Services; MACPAC, Medicaid and CHIP Payment Access Commission; NADAC, National Average Drug Acquisition Costs; WAC, wholesale acquisition cost. Prices were normalized for product strength based on authors’ calculations of list and net prices using data from SSR Health and commercial pharmacy claims; net expenditure calculations also incorporate data from Drug Channels Institute, Medicaid MACPAC reports, Medicare Part D claims, CMS NADAC, Ohio Medicaid Auditor of State report, and Nevada State Drug Transparency report.

Figure 2 illustrates trends in 3 key insulin cost measures: (1) the undiscounted list price (or WAC) reported in many media stories, (2) the net price received by manufacturers after discounts and rebates to distribution system participants, and (3) the systemwide net insulin expenditures, which include the net price to manufacturers plus gross profits of all distribution system participants. Per 100 units of insulin, list price increased substantially from $19.60 in 2014 to $27.45 in 2018, representing a 40.1% increase over 5 years. In contrast, the net price decreased 30.8%, from $10.53 in 2014 to $7.29 in 2018. The difference between list and net prices increased over time by 122.5%, from $9.06 per 100 units of insulin in 2014 to $20.16 in 2018. Net expenditure per 100 units of insulin has changed little, increasing just 3.2% from $15.11 in 2014 to $15.59 in 2018.

### Allocation of Expenditures Across Distribution System Participants

**Figure 3.**
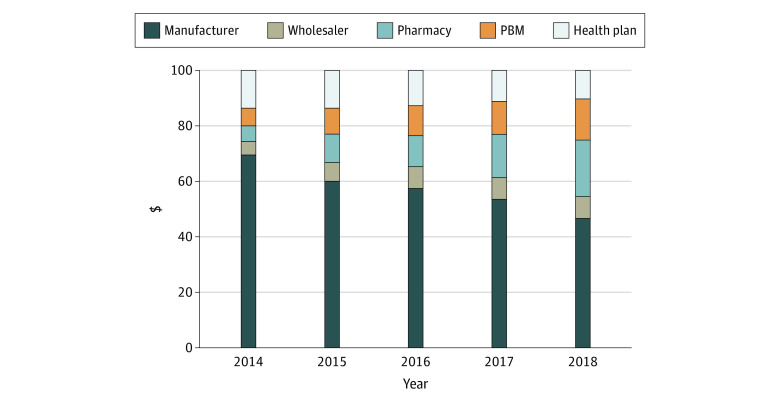
Average Distribution of $100 in Insulin Expenditures for 32 Insulin Products Across Distribution System Participants, 2014-2018 Abbreviations: CMS, Centers for Medicare & Medicaid Services; MACPAC, Medicaid and CHIP Payment Access Commission; NADAC, National Average Drug Acquisition Costs; PBM, pharmacy benefit manager. The insulin products were normalized for product strength. All analyses were based on authors’ calculations of data from SSR Health, Medicaid MACPAC reports, commercial and Medicare Part D pharmacy claims, CMS NADAC, Drug Channels Institute reports, Ohio Medicaid Auditor of State report, and Nevada State Drug Transparency report.

Although expenditures per 100 units of insulin changed little, their composition changed notably during this period. Figure 3 depicts the distribution of a hypothetical $100 in net expenditures on insulin across manufacturers and other distribution system participants each year. The shares of manufacturers and insurers have decreased between 2014 and 2018, from $69.71 to $46.73 (−33.0%) for manufacturers, and from $13.82 to $10.40 (−24.7%) for insurers. In contrast, the amount retained by wholesalers, pharmacies, and PBMs has increased notably over that period, from $4.63 to $8.09 (74.7%) for wholesalers, from $6.21 to $20.42 (228.8%) for pharmacies, and from $5.64 to $14.36 (154.6%) for PBMs. Overall, the share of a hypothetical $100 insulin expenditure that accrued to manufacturers decreased such that by 2018 more than half of expenditures on insulin flowed to distribution system intermediaries. eFigure 1 in the Supplement presents results of the same analysis per 100 mL of insulin, that is, without normalizing dollars spent. Subcategory analysis of long-acting vs non–long-acting insulins and vials vs pens is presented in eFigure 2 in the Supplement and shows similar trends.

### Sensitivity Analyses

To understand how imprecision in parameter estimates may be a factor in our results, we conducted sensitivity analyses varying key parameter values (the share of the gross-to-net reduction going to rebates, insulin net price, PBM spread, insulin OOP payments, the distribution of manufacturer fees and discounts between PBMs and pharmacies, the share of rebates retained by PBMs, health plan gross margins, and the inclusion of biosimilar insulin) and observed changes in the resulting distribution of expenditures across industry participants. While specific outcome values changed as parameters varied, the overall result of decreasing manufacturer and health plan shares and increasing shares for distribution system intermediaries over time was unchanged. eTables 5 and 6 in the Supplement detail how parameters were varied in the sensitivity analyses, and eTable 7 in the Supplement contains the results of the sensitivity analyses.

## Discussion

Policy makers and health policy experts often blame manufacturers for increasing insulin costs, citing the market power of 3 companies accounting for 90% of the market.^27,28^ Results of this analysis demonstrate how all entities in the pharmaceutical distribution system (manufacturers, wholesalers, pharmacies, PBMs, and health plans) profit from the sale of insulin and that all contribute to its final price. A more complete and dynamic picture of the contribution of each entity to insulin expenditures may help guide effective policy development.

We estimated a widening gap between list and net prices between 2014 and 2018, suggesting that manufacturers offered increasing discounts and rebates to distribution system intermediaries. Despite the manufacturer net prices decreasing in every year, net expenditures per 100 units of insulin were consistent, increasing only 3.2% in 5 years. Competitive pressure from the 2017 introduction of a biosimilar insulin glargine injectable (Basaglar; Eli Lilly) may have contributed to the decrease in net price.

The widening gap between net expenditures and net prices suggests that increasing profits of distribution system participants are responsible for the increase in insulin costs. While we estimate that proceeds from insulin sales flowing to insulin manufacturers and insurers have decreased over time, those flowing to PBMs, pharmacies, and wholesalers have increased substantially, and in 2018 accounted for 42.9% of all expenditures, compared with 16.5% in 2014. The PBM and wholesaler markets are also highly concentrated; in 2018, the 3 largest PBMs accounted for more than 70% of all prescription claims, and the top 3 wholesalers accounted for 95% of distribution revenues.^29,30^ Subcategory analysis shows that intermediaries captured a larger share of expenditures on long-acting than short-acting and intermediate-acting products and more on pens than vials.

While focusing solely on manufacturers and PBMs, the recent Senate Finance Committee report^11^ on insulin reached similar conclusions, finding that PBMs were complicit in efforts to raise prices and rebates, sometimes discouraging manufacturers from lowering insulin prices. The report also emphasized the need to bring greater transparency to pharmaceutical pricing and marketing in efforts to reduce drug costs.^11^

Recently, CMS introduced a demonstration model for 2021 that would lower OOP costs for insulin to a maximum $35 copay per 30-day supply for beneficiaries in participating plans.^31^ While limiting beneficiaries’ OOP costs may provide immediate financial relief to insulin users, it does not fundamentally resolve the problem of increasing insulin costs, and such proposals could create more pressure on premiums to increase. Instead, we recommend a solution that addresses the root cause of increasing insulin costs, particularly addressing the roles played by distribution system intermediaries.

### Limitations

This study has several limitations. Data come from a variety of sources covering sometimes incomplete time series, different patient populations, and different payers and market segments with varying benefit designs and rebate amounts, and have been assembled using different methods. We used SSR Health data to estimate gross-to-net reductions at the product level. Others have found that SSR data may overestimate discounts,^32^ but these data have been used in other peer-reviewed literature^33^ as a source for product-level discounts.

While we have used parameter estimates specific to insulin whenever available, when missing, we used estimates based on all diabetes drugs, or, lacking those, on all brand-name drugs. Although sensitivity analyses suggest the overall result of decreasing manufacturer and health plan shares combined with increasing intermediary shares is robust to uncertainty in key parameter values, our results are best interpreted as suggesting trends in insulin markets generally rather than as providing a precise picture of a particular insulin market. While there are good data showing that the gross-to-net reduction has increased, we lack complete time-series data on transaction prices at each step of the distribution system, and therefore the split of excess earnings among intermediaries may be imprecise. For example, the share of manufacturer payments going to pharmacies vs PBMs may have decreased over 2014-2018, but without data to support a time trend, we have assumed the share remains constant, which may lead us to overestimate the share taken by pharmacies and underestimate that taken by PBMs. Although there is robust evidence showing increasing excess earnings to distribution intermediaries taken together, incomplete data at each transaction within the system mean the distribution we find among intermediaries should be interpreted with caution. Even with these limitations, our research suggests a need for greater transparency in drug pricing, which could help resolve many of the data limitations we have faced.

## Conclusions

In this cross-sectional study of the distribution of insulin expenditures, we suggest that policy makers looking for ways to control insulin costs may wish to consider entities throughout the insulin distribution system instead of only manufacturers. Findings of our analysis further suggest that manufacturers represented a decreasing share of insulin expenditures, whereas PBMs, pharmacies, and wholesalers accounted for an increasing share. Insulin manufacturers, PBMs, and wholesalers operate in highly concentrated markets in which market power is a concern.

## Notes

Supplement. **eFigure 1.** Distribution of Net Expenditures per 100 Units of Insulin Across Distribution System Participants, 2014-2018 **eFigure 2.** Distribution of $100 in Expenditures Across Insulin Distribution System Participants, Insulin Sub-categories (Normalized for Product Strength), 2014-2018 **eTable 1.** List of Insulin Products Included in Analysis **eTable 2.** Key Data Sources and Their Underlying Sources **eMethods.** Methods to Allocate Gross-to-Net Reductions Across Pharmaceutical Supply Chain Entities **eTable 3.** Decomposition of Gross-to-Net Reduction Into Rebates and Copay Offset Programs ($billions) **eTable 4.** Distribution of Fees and Discounts From Manufacturers Among Drug Channel Participants **eTable 5.** Summary Percentages of Gross-to-Net Reduction Into Rebates, Copay Offset Programs and Fees and Discounts—Baseline and Sensitivity Analysis Scenarios 1a and 1b **eTable 6.** Distribution of Fees and Discounts From Manufacturers Among Drug Channel Participants—Baseline and Sensitivity Analysis Scenarios 5a and 5b **eTable 7.** Results of Sensitivity Analyses: Distribution of $100 in Expenditures Across Insulin Distribution System Participants, 32 Insulin Products (Normalized for Product Strength), 2014-2018
eReferences
