# Supplementary material for: Estimation of the Share of Net Expenditures on Insulin Captured by US Manufacturers, Wholesalers, Pharmacy Benefit Managers, Pharmacies, and Health Plans From 2014 to 2018
Source: JAMA Health Forum. 2021 Nov 5;2(11):e213409. doi: 10.1001/jamahealthforum.2021.3409 (PMC8796876; doi:10.1001/jamahealthforum.2021.3409)
Supplement: Supplement. — eFigure 1. Distribution of Net Expenditures per 100 Units of Insulin Across Distribution System Participants, 2014-2018 eFigure 2. Distribution of $100 in Expenditures Across Insulin Distribution System Participants, Insulin Sub-categories (Normalized for Product Strength), 2014-2018 eTable 1. List of Insulin Products Included in Analysis eTable 2. Key Data Sources and Their Underlying Sources eMethods. Methods to Allocate Gross-to-Net Reductions Across Pharmaceutical Supply Chain Entities eTable 3. Decomposition of Gross-to-Net Reduction Into Rebates and Copay Offset Programs ($billions) eTable 4. Distribution of Fees and Discounts From Manufacturers Among Drug Channel Participants eTable 5. Summary Percentages of Gross-to-Net Reduction Into Rebates, Copay Offset Programs and Fees and Discounts—Baseline and Sensitivity Analysis Scenarios 1a and 1b eTable 6. Distribution of Fees and Discounts From Manufacturers Among Drug Channel Participants—Baseline and Sensitivity Analysis Scenarios 5a and 5b eTable 7. Results of Sensitivity Analyses: Distribution of $100 in Expenditures Across Insulin Distribution System Participants, 32 Insulin Products (Normalized for Product Strength), 2014-2018 eReferences [file jamahealthforum-e213409-s001.pdf]

Supplemental Online Content

Van Nuys K, Ribero R, Ryan M, Sood N. Estimation of the share of net expenditures on insulin captured by US manufacturers, wholesalers, pharmacy benefit managers, pharmacies, and health plans from 2014 to 2018. *JAMA Health Forum*. 2021;2(11):e213409. doi:10.1001/jamahealthforum.2021.3409

eFigure 1. Distribution of Net Expenditures per 100 Units of Insulin Across Distribution System Participants, 2014-2018

eFigure 2. Distribution of $100 in Expenditures Across Insulin Distribution System Participants, Insulin Sub-categories (Normalized for Product Strength), 2014-2018

eTable 1. List of Insulin Products Included in Analysis

eTable 2. Key Data Sources and Their Underlying Sources

eTable 3. Decomposition of Gross-to-Net Reduction Into Rebates and Copay Offset Programs ($billions)

eTable 4. Distribution of Fees and Discounts From Manufacturers Among Drug Channel Participants

eTable 5. Summary Percentages of Gross-to-Net Reduction Into Rebates, Copay Offset Programs and Fees and Discounts – Baseline and Sensitivity Analysis Scenarios 1a and 1b

eTable 6. Distribution of Fees and Discounts From Manufacturers Among Drug Channel Participants – Baseline and Sensitivity Analysis Scenarios 5a and 5b

eTable 7. Results of Sensitivity Analyses: Distribution of $100 in Expenditures Across Insulin Distribution System Participants, 32 Insulin Products (Normalized for Product Strength), 2014-2018

eReferences

This supplemental material has been provided by the authors to give readers additional information about their work.

© 2021 Van Nuys K et al. *JAMA Health Forum.*

eFigure 1. Distribution of Net Expenditures per 100 Units of Insulin Across Distribution System Participants 2014-2018

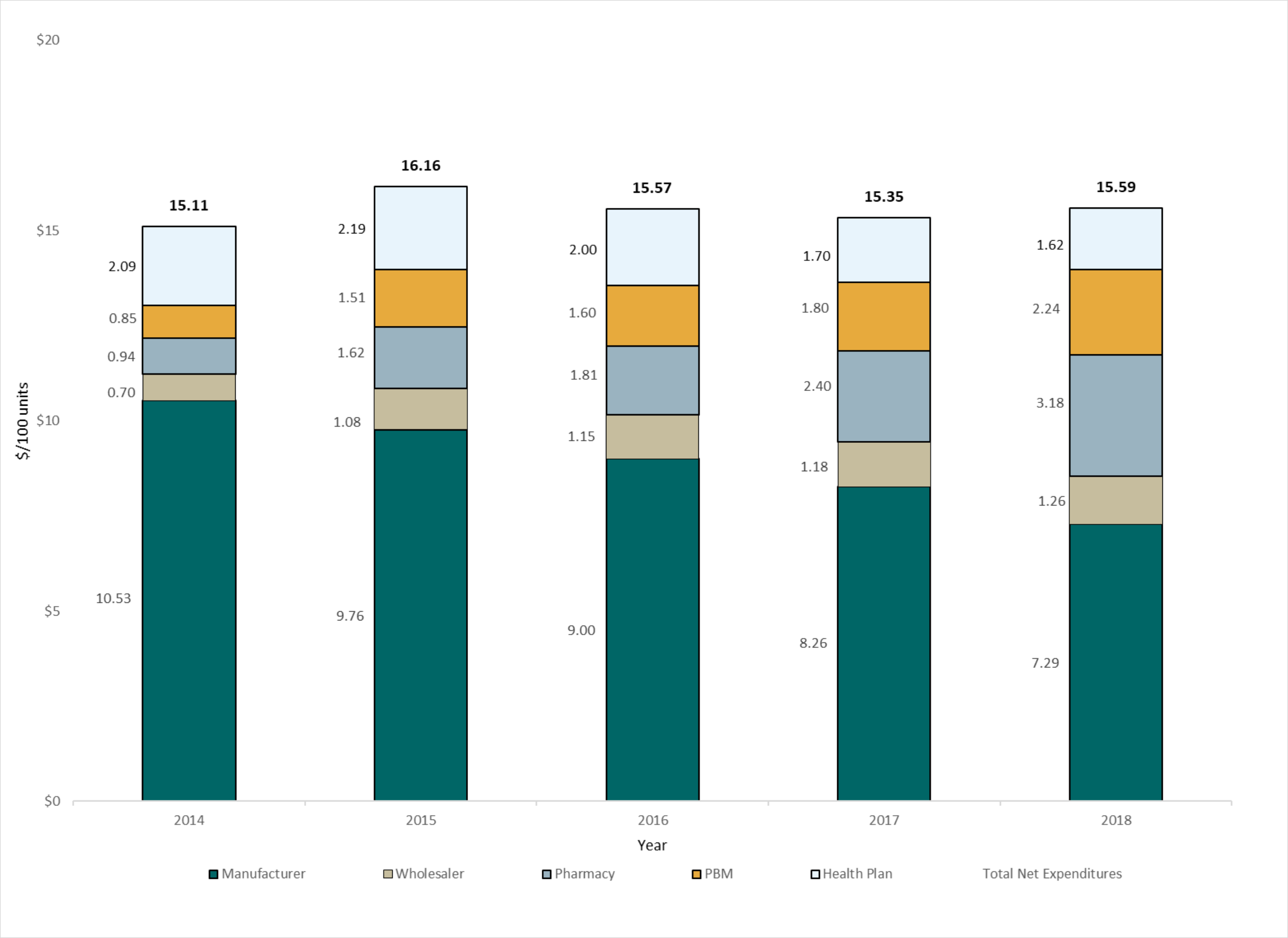

Notes:

32 insulin products included, normalized for product strength, measured in $/100 units of insulin.

© 2021 Van Nuys K et al. *JAMA Health Forum.*

eFigure 2: Distribution of $100 in Expenditures Across Insulin Distribution System Participants, Insulin Sub-categories (normalized for product strength), 2014-2018

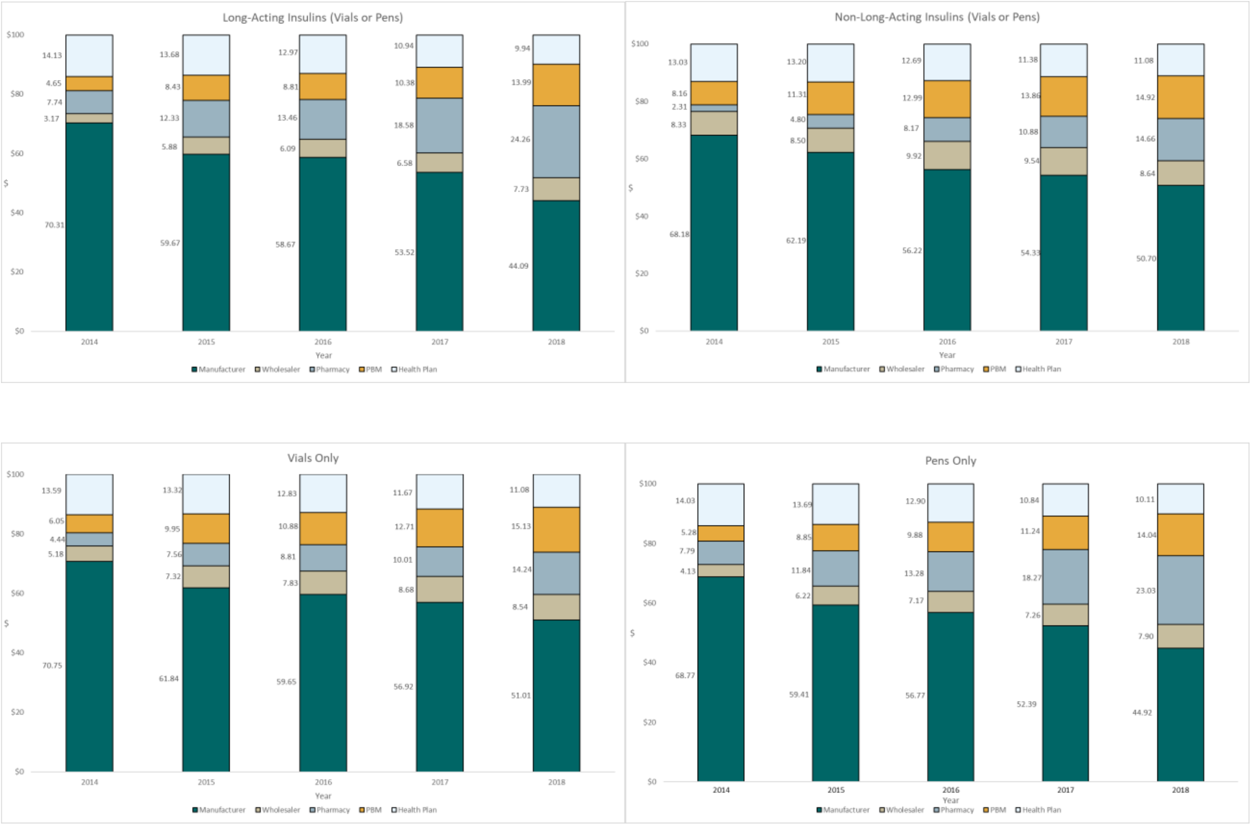

© 2021 Van Nuys K et al. *JAMA Health Forum.*

eTable1. List of Insulin Products Included in Analysis

(except where noted, all products contain 100 units of insulin per mL)

		Year							
**NDC**	**Brand Name**	**2014**	**2015**	**2016**	**2017**	**2018**	**2014-2018**	**Long-Acting**	**Pen**
00088250033	Apidra	X	X	X	X	X	X		
00088250205	Apidra Solostar	X	X	X	X	X	X		X
00002771559	Basaglar Kwikpen U-100				X	X	X	X	X
00169320111	Fiasp					X	X		
00169320415	Fiasp Flextouch					X	X		X
00002751001	Humalog	X	X	X	X	X	X		
00002751017	Humalog	X	X	X	X	X	X		
00002771459	Humalog Junior Kwikpen					X	X		X
00002879959	Humalog Kwikpen U-100	X	X	X	X	X	X		X
00002771227	Humalog Kwikpen U-200[1]		X	X	X	X	X		X
00002751201	Humalog Mix 50-50	X	X	X	X	X	X		
00002751101	Humalog Mix 75-25	X	X	X	X	X	X		
00002871501	Humulin 70-30	X	X	X	X	X	X		
00002871517	Humulin 70-30	X	X	X	X	X	X		
00002877059	Humulin 70-30	X	X				X		
00002880359	Humulin 70/30 Kwikpen	X	X	X	X	X	X		X
00002831501	Humulin N	X	X	X	X	X	X		
00002831517	Humulin N	X	X	X	X	X	X		
00002873059	Humulin N	X	X				X		
00002880559	Humulin N Kwikpen	X	X	X	X	X	X		X
00002821501	Humulin R	X	X	X	X	X	X		
00002821517	Humulin R	X	X	X	X	X	X		
00002850101	Humulin R U-500[2]	X	X	X	X	X	X		
00002882427	Humulin R U-500 Kwikpen[2]			X	X	X	X		X
00088222033	Lantus	X	X	X	X	X	X	X	
00088502101	Lantus				X	X	X	X	
00088221905	Lantus Solostar	X	X	X	X	X	X	X	X
00088502005	Lantus Solostar					X	X	X	X
00169368712	Levemir	X	X	X	X	X	X	X	
00169643910	Levemir Flexpen	X	X	X			X	X	X
00169643810	Levemir Flextouch	X	X	X	X	X	X	X	X
00169183702	Novolin 70-30	X	X	X	X	X	X		
00169183711	Novolin 70-30	X	X	X	X	X	X		
00169183402	Novolin N	X	X	X	X	X	X		
00169183411	Novolin N	X	X	X	X	X	X		
00169183302	Novolin R	X	X	X	X	X	X		
00169183311	Novolin R	X	X	X	X	X	X		
00169750111	Novolog	X	X	X	X	X	X		
00169368512	Novolog Mix 70-30	X	X	X	X	X	X		
00024587102	Toujeo Max Solostar[3]					X	X	X	X
00024586903	Toujeo Solostar[3]		X	X	X	X	X	X	X
00169266015	Tresiba Flextouch U-100			X	X	X	X	X	X
00169255013	Tresiba Flextouch U-200[1]			X	X	X	X	X	X

© 2021 Van Nuys K et al. *JAMA Health Forum.*

		Year							
**NDC**	**Brand Name**	**2014**	**2015**	**2016**	**2017**	**2018**	**2014-2018**	**Long-Acting**	**Pen**
	Count of NDCs	31	33	34	35	40	43		
	Count of Brand Names	22	24	27	27	31	32		

Notes:

X indicates that the product data was used in year
NDC: National Drug Code
[1]Product strength is 200 units per mL.
[2]Product strength is 500 units per mL.
[3]Product strength is 300 units per mL.

© 2021 Van Nuys K et al. *JAMA Health Forum.*

Data inputs and their sources

Our analysis relies on data from multiple sources, including Drug Channels Institute, a consulting firm specializing in the economics of the pharmaceutical distribution industry that publishes annual reports with industry data gathered from many other sources. eTable2 lists those inputs, the sources from which we took them, and those sources' source:

eTable2. Key Data Sources and Their Underlying Sources

Analysis Input	Source	Source's Source	Notes
1. Total gross-to-net reduction on all brand drugs	DCI [3] Exhibit 159	IQVIA Institute	IQVIA is a data vendor contracted by the government to collect data measuring the volume of pharmaceuticals sold by manufacturers and wholesalers to pharmacies, hospitals and other settings
2. Medicaid rebates	DCI [3] Exhibit 156	MACStats: Medicaid and CHIP Data Book, MACPAC	Source data from multiple years of government Medicaid program reports, assembled by DCI
3. Medicare part D rebates	CMS Part D Rebate Summary for All Brand Name Drugs, 2014 [1]<br>DCI [3] Exhibit 154<br>DCI blog post from April 3, 2019 [2]	CMS Manufacturer Rebate Summary<br>CMS and US GAO<br>IQVIA	Government data<br>Government data<br>Data vendor contracted by government (see #1 above)
4. Commercial payer rebates	DCI [3] Exhibit 160<br>DCI blog post from April 3, 2019 [2]z	Employer surveys, state regulatory filings, PBM financial disclosures<br>IQVIA<br>IQVIA	DCI uses proprietary methodology to combine data from different sources to estimate the fraction of the gross-to-net reduction going to commercial rebates<br>Data vendor contracted by government (see #1 above)
5. Proportion of Gross to Net difference represented by rebates	DCI (see items 1-4 above)	See items 1-4 above	Government and IQVIA data
6. Spending on patient assistance and copay support programs	DCI [3] Exhibit 114	IQVIA	Data vendor contracted by government (see #1 above)
7. Share of fees and discounts from manufacturers going to wholesalers	SEC filings of largest three wholesalers	Company calculations	
8. Share of fees and discounts from manufacturers going to PBMs	Assumption		Assumed remainder split evenly; test the impact of that assumption in sensitivity analysis below

© 2021 Van Nuys K et al. *JAMA Health Forum.*

9. Share of fees and discounts from manufacturers going to pharmacies	Assumption		Assumed remainder split evenly; test the impact of that assumption in sensitivity analysis below
10. Out of Pocket Expenditure	Medicare VRDC	Medicare Claims	
11. Spread payment	Ohio Auditor of State Report	Audit of Ohio Managed Medicaid program	
12. Net and list prices of insulins, by NDC	SSR Health	Manufacturer financial statements and earnings calls, Symphony Health	
13. Share of rebates kept by PBMs	Nevada transparency report	State survey responses by PBMs	
14. Insurer gross margins	SEC filings of 10 largest publicly traded insurers	10K and 20F reports	

Methods to allocate gross-to-net reductions across pharmaceutical supply chain entities

In this section we describe the data sources and methods used to allocate total gross-to-net reductions or gross-to-net bubble into the amounts represented by flows 2-6 in Figure 1 of the manuscript.

We begin with the total gross-to-net reductions reported by Drug Channels Institute's (DCI) annual Economic Report on U.S. Pharmacies and Pharmacy Benefit Managers in the first row of eTable3.[3]

We decompose the total gross-to-net reduction into its constituent parts, using data published by DCI.[3] These data are not available to us in every year, so we use additional sources and methods, as detailed below, to estimate values in missing years.

Following the DCI methodology, we decompose the gross-to-net reduction into five components: (a) spending on patient assistance and copayment support, (b) Medicaid rebates, (c) Medicare Part D rebates, (d) commercial payer rebates, and (e) fees and discounts from manufacturers to drug channel participants, including discounts to providers under the 340B pricing program.

(a) Patient Assistance and Copayment Support

To calculate the total amount spent on patient assistance and copayment support for years 2014 to 2019, we used Exhibit 114 from the 2020 DCI Report ("Manufacturer Spending on Copay Offset Programs from 2014 to 2019") which estimates the value for 2019.[3] These values are reported in row A of eTable3.

For the remaining components, the data needed to decompose the gross-to-net reduction were only available from DCI in some years, so we augmented with data from other sources as follows:

(b) Medicaid Rebates

© 2021 Van Nuys K et al. *JAMA Health Forum.*

For Medicaid Rebates in 2015-18 we used the values in Exhibit 156 of the 2020 DCI report ("Medicaid Gross v. Net Spending on Outpatient Drugs from 2015 – 2018").[3] For 2014, we used the MACPAC report "Medicaid Drug Spending Trends February 2019."[4] For 2019 we used Exhibit 160 from the DCI report ("Total Value of Pharmaceutical Manufacturers' Gross-to-Net Reductions for Brand-name Drugs, by Source"), and calculated Medicaid Rebates as 18% of the Gross-to-Net Reduction in 2019. These values are reported in row B of eTable3.

(c) Medicare Part D Rebates

For the Medicare Part D rebates we used the $16.3 billion figure from CMS' 2014 Part D Rebate Summary for All Brand Name Drugs, [1] and the 2019 figure of $40.0 billion from Exhibit 154 of the 2020 DCI Report ("Medicare Part D, Direct and Indirect Remuneration (DIR), by Source, 2013 vs. 2019").[3] For years 2015, 2016 and 2018 we assumed constant growth between 2014 and 2019, and for 2017 we used DCI data from the blog post "The Gross-to-Net Bubble Reached a Record $166 Billion in 2018" from April 3, 2019 to calculate 19% of the gross-to-net difference, or $29.1 billion, in Medicare Part D rebates in 2017.[2] These values are reported in row C of eTable3.

(d) Commercial Payer Rebates

To calculate commercial payer rebates we used the commercial rebate share given in DCI's April 3, 2019 blog post for 2017 [2] and the share given in Exhibit 160 of the 2020 DCI report for 2019,[3] and applied them to the gross-to-net reduction in Row A of eTable3. We impute the value for 2018 as the arithmetic average of the 2017 and 2019 values; the values for 2014-2016 are imputed to result in constant growth over time. These values are reported in row D of eTable3.

(e) Fees and Discounts from Manufacturers to Drug Channel Participants

We calculated the other fees and discounts from manufacturers to drug channel participants as the difference between the total gross-to-net reductions and the other four elements calculated above: (a) manufacturer spending on copay offset programs, (b) Medicaid rebates, (c) Medicare Part D rebates, and (d) commercial payer rebates. These values are reported in row G of eTable3.

(See eTable3)

Using the values in eTable3, we calculated the share of the total gross-to-net reduction that each component represented in each year and applied that share to the product-specific gross-to-net reductions for insulins, calculated from SSR data in each year to calculate the magnitude of the flows 2-6 in Exhibit 1 of the main manuscript. To calculate the shares, we relied on estimates from a common source (DCI) for both the denominator and the numerator of the estimate. But while we rely on DCI estimates of the aggregate gross-to-net reduction to calculate the shares in eTable3, we apply those shares to product-specific estimates of the gross-to-net reductions from SSR; DCI does not provide gross-to-net reduction estimates at the product level.

The values for manufacturer spending on copay offset programs (row A) correspond to flow 3, total rebates (row E) correspond to flow 2, and fees and discounts from manufacturers to drug channel participants (row G) correspond to the sum of flows 4, 5 and 6 in Exhibit 1 of the manuscript.

To estimate how the amounts in row G are allocated into individual shares for flows 4, 5 and 6 (fees and discounts to wholesalers, PBMs and pharmacies, respectively), we used the gross profit margins

© 2021 Van Nuys K et al. *JAMA Health Forum.*

reported in the financial statements of the three largest wholesalers for each year, and adjusted the model parameters so that the average gross profit margin of the wholesalers in our model match the observed margin of the wholesalers in each year. We assumed that the remaining share of discounts and fees is distributed evenly among pharmacies and PBMs.[5]-[10] The resulting shares are reported in eTable4.

(See eTable4)

© 2021 Van Nuys K et al. *JAMA Health Forum.*

eTable3. Decomposition of Gross-to-Net Reduction into Rebates and Copay Offset Programs ($billions)

	Year					
	**2014**	**2015**	**2016**	**2017**	**2018**	**2019**
Total Gross-to-Net Reduction[a]	102	124	139	153	166	175
A. Manufacturer Spending on Copay Offset Programs[b]	6	8	10	11	13	15
A as a % of Gross-to-Net Reduction	6%	6%	7%	7%	8%	9%
B. Medicaid Rebates[c]	19.9	24.0	31.2	34.9	36.2	31.5
B as a % of Gross-to-Net Reduction	20%	19%	22%	23%	22%	18%
C. Medicare Part D Rebates[d]	16.3	*21.0*	*25.8*	29.1	*35.3*	40.0
C as a % of Gross-to-Net Reduction	16%	17%	19%	19%	21%	23%
D. Commercial Payer Rebates[e]	*35.0*	*37.1*	*39.2*	41.3	*43.4*	45.5
D as a % of Gross-to-Net Reduction	34%	30%	28%	27%	26%	26%
E. Total Rebates (Subtotal B + C +D)	71.2	82.2	96.2	105.3	114.9	117.0
E as % of Total Gross to Net Reduction	70%	66%	69%	69%	69%	67%
F. Sum A+B+C+D	77.2	90.2	106.2	116.3	127.9	132.0
F as a % of Gross-to-Net Reduction	76%	73%	76%	76%	77%	75%
G. Fees and Discounts from Manufacturers to Drug Channel Participants[f,g]	24.8	33.8	32.8	36.7	38.1	43.0
G as a % of Gross-to-Net Reduction	24%	27%	24%	24%	23%	25%

Legend:

Italics = data were imputed based on authors calculations/projections.

Sources and notes:

[a] Data from Exhibit 159 ("Total Value of Pharmaceutical Manufacturers' Gross-to-Net Reductions for Brand Name Drugs, 2014 to 2019") in The 2020 Economic Report on U.S. Pharmacies and Pharmacy Benefit Managers, Drug Channels Institute, 2020.[3]

[b] Values for all years from Exhibit 114 ("Manufacturer Spending on Copay Offset Programs") from 2020 DCI Report.[3]

© 2021 Van Nuys K et al. *JAMA Health Forum.*

[c] 2014 data from MACPAC Medicaid Drugs Spending Trends 2019 [4]; 2015-18 data from Exhibit 156 ("Medicaid, Gross vs. Net Spending on Outpatient Drugs, 2015 - 2018") of 2020 DCI Report [3]; 2019 data calculated as 18% of the $175b gross-to-net reduction in 2019, where 18% is taken from Exhibit 160 of the same report.
[d] 2019 data from Exhibit 154 ("Medicare Part D, Direct and Indirect Remuneration (DIR), by Source, 2013 vs. 2019") of DCI 2020 report.[3] 2014 data from https://www.cms.gov/Research-Statistics-Data-and-Systems/Statistics-Trends-and-Reports/Information-on-Prescription-Drugs/PartD_Rebates#1-note. Values for 2015, 2016 and 2018 imputed assuming constant growth between 2014 and 2019; data for 2017 calculated as 19% of the $153b gross-to-net reduction in 2017, where 19% is taken from DCI Blog post of April 3, 2019.[2]
[e] Value for 2017 is computed by applying the 27% commercial rebate percentage from DCI Blog post of April 3, 2019 to the 2017 total gross-to-net reduction; value for 2019 is computed by applying the 26% commercial rebate percentage from Exhibit 160 in the 2020 DCI Report. Value for 2018 imputed by applying the average commercial rebate from 2017 and 2019. Values for 2014, 2015 and 2016 are imputed to deliver a constant growth rate throughout the period.
[f] Computed as Total Gross-to-Net Reduction minus Sum A + B + C + D.
[g] Some totals or percentages may not add due to rounding of figures by authors or in sources.

eTable4. Distribution of Fees and Discounts from Manufacturers Among Drug Channel Participants

	Year				
	**2014**	**2015**	**2016**	**2017**	**2018**
Discount from list prices to wholesaler (adjusted to fit Wholesaler Gross Margins from financial statements)	60%	48%	51%	50%	50%
Fees and discounts from manufacturers to drug channel participants-PBM	20%	26%	24%	25%	25%
Fees and discounts from manufacturers to drug channel participants-pharmacy	20%	26%	24%	25%	25%

© 2021 Van Nuys K et al. *JAMA Health Forum.*

Sensitivity analyses

Because our analysis relies on parameter estimates from a wide range of sources with varying precision, we explored the impact of changes in those parameters, to understand how uncertainty in those estimates may affect our results. eTable7 presents the results of varying key parameters in 8 scenarios:

Scenario 1a,b: Vary the share of gross-to-net reductions going to rebates (see row E of eTable3 above) by +/-10%. To fully allocate the gross-to-net reduction across rebates, copay assistance programs and other fees and discounts, we simultaneously changed the remaining share going to the last two categories, as reflected in eTable5, so that the shares sum to 100% in each year.

Scenario 2a,b: Vary the net prices of insulins in our sample by +/-10%.

Scenario 3a,b: Vary the spread that PBMs keep (the difference between what health plans pay PBMs and what PBMs pay pharmacies) by +/-50%.

Scenario 4: Use commercial claims instead of Medicare claims to calculate the average patient out-of-pocket payment on insulin claims.

Scenario 5a,b: Assume that, after wholesaler discounts, remaining fees and discounts are split 25%/75% (Scenario 5a) or 75%/25% (Scenario 5b) between PBMs and pharmacies (see eTable6).

Scenario 6a,b,c: Assume PBMs pass (92.5%/90%/87.5%) of rebates to health plans.

Scenario 7a,b: Vary gross margin of health plans by +/-10%.

Scenario 8: Exclude biosimilar insulin glargine injection (Basaglar) from analyses.

© 2021 Van Nuys K et al. *JAMA Health Forum.*

eTable5. Summary Percentages of Gross-to-Net Reduction into Rebates, Copay Offset Programs and Fees and Discounts – Baseline and Sensitivity Analysis Scenarios 1a and 1b

	Year				
	**2014**	**2015**	**2016**	**2017**	**2018**
**Baseline (See eTable3 Row E)**					
Manufacturer Spending on Copay Offset Programs as a % of Gross-to-Net Reduction	6%	6%	7%	7%	8%
Fees and Discounts from Manufacturers to Drug Channel Participants as a % of Gross-to-Net Reduction	24%	27%	24%	24%	23%
Rebates as a % of Gross-to-Net Reduction	70%	66%	69%	69%	69%
**Scenario 1a: Increase Rebate Share 10%**					
Manufacturer Spending on Copay Offset Programs as a % of Gross-to-Net Reduction	5%	5%	6%	6%	6%
Fees and Discounts from Manufacturers to Drug Channel Participants as a % of Gross-to-Net Reduction	19%	22%	18%	19%	18%
Rebates as a % of Gross-to-Net Reduction	77%	73%	76%	76%	76%
**Scenario 1b: Decrease Rebate Share 10%**					
Manufacturer Spending on Copay Offset Programs as a % of Gross-to-Net Reduction	7%	8%	9%	9%	10%
Fees and Discounts from Manufacturers to Drug Channel Participants as a % of Gross-to-Net Reduction	30%	33%	29%	29%	28%
Rebates as a % of Gross-to-Net Reduction	63%	60%	62%	62%	62%

© 2021 Van Nuys K et al. *JAMA Health Forum.*

eTable6 Distribution of Fees and Discounts from Manufacturers Among Drug Channel Participants—Baseline and Sensitivity Analysis Scenarios 5a and 5b

	Year				
	2014	2015	2016	2017	2018
**Baseline (see eTable4)**					
Discount from list prices to wholesaler (adjusted to fit Wholesaler Gross Margins from financial statements)	60%	48%	51%	50%	50%
Fees and discounts from manufacturers to drug channel participants-PBM	20%	26%	24%	25%	25%
Fees and discounts from manufacturers to drug channel participants-pharmacy	20%	26%	24%	25%	25%
**Scenario 5a: Distribute remaining share after wholesaler discounts 75/25% to pharmacies/PBMs**					
Discount from list prices to wholesaler (adjusted to fit Wholesaler Gross Margins from financial statements)	60%	48%	51%	50%	50%
Fees and discounts from manufacturers to drug channel participants-PBM	10%	12.9%	12.1%	12.4%	12.4%
Fees and discounts from manufacturers to drug channel participants-pharmacy	30%	38.7%	36.4%	37.2%	37.3%
**Scenario 5b: Distribute remaining share after wholesaler discounts 25/75% to pharmacies/PBMs**					
Discount from list prices to wholesaler (adjusted to fit Wholesaler Gross Margins from financial statements)	60%	48%	51%	50%	50%
Fees and discounts from manufacturers to drug channel participants-PBM	30%	38.7%	36.4%	37.2%	37.3%
Fees and discounts from manufacturers to drug channel participants-pharmacy	10%	12.9%	12.1%	12.4%	12.4%

© 2021 Van Nuys K et al. *JAMA Health Forum.*

eTable7 Results of Sensitivity Analyses. Distribution of $100 in Expenditures Across Insulin Distribution System Participants, 32 Insulin Products (normalized for product strength), 2014-2018

		% change in share, 2014-18 going to					
**Simulation Description**	**Mfrs' Share of $100 in Insulin Expend-itures, 2018**	**Mfrs**	**Whole-salers**	**Phar-macies**	**PBMs**	**Health Plans**	**Key Result vs. Baseline**
**Baseline**	**46.73**	**-33%**	**75%**	**229%**	**155%**	**-25%**	---
**Scenario 1a:** Increase share of gross-to-net reduction going to rebates by 10%	51.92	-29%	91%	271%	172%	-28%	Manufacturers keep slightly more (less) as wholesaler and pharmacy shares decrease (increase) when share of gross-to-net going to rebates increases (decreases). Small changes in PBM and health plan shares.
**Scenario 1b:** Decrease share of gross-to-net reduction going to rebates by 10%	42.48	-36%	64%	202%	140%	-22%	
**Scenario 2a:** Increase net prices of insulins by 10%	49.61	-32%	113%	270%	179%	-25%	Increasing (decreasing) net prices reduces (enlarges) the pool from which intermediaries take their share, so it decreases (increases), while manufacturers' share increases (decreases).
**Scenario 2b:** Decrease net prices of insulins by 10%	43.63	-34%	50%	196%	134%	-25%	
**Scenario 3a:** Increase PBM spread by 50%	43.63	-34%	75%	228%	146%	-25%	Manufacturer and health plan shares largely unchanged; minor redistribution of rents among intermediaries, with slightly more (less) going to PBMs and less (more) to pharmacies and wholesalers as PBM spread increases (decreases).
**Scenario 3b:** Decrease PBM spread by 50%	47.03	-33%	75%	229%	164%	-25%	
**Scenario 4:** Estimate mean OOP payments on insulins from commercial rather than Medicare claims	44.71	-34%	72%	223%	149%	-11%	Commercial OOP lower than Medicare. Health plan share is larger but grows more slowly. Other players' shares largely unchanged.
**Scenario 5a:** After wholesaler discounts, distribute remaining	46.73	-33%	75%	212%	154%	-25%	Shares to manufacturers, wholesalers and health plans unchanged. Shares to PBMs

© 2021 Van Nuys K et al. *JAMA Health Forum.*

fees 75% to pharmacies, 25% to PBMs							decrease (increase), while shares to pharmacies increase (decrease) commensurately.
**Scenario 5b:** After wholesaler discounts, distribute remaining fees 25% to pharmacies, 75% to PBMs	46.73	-33%	75%	252%	154%	-25%	
**Scenario 6a:** PBMs Retain 7.5% of Insulin Rebates	46.28	-33%	76%	231%	122%	-25%	PBM share increases, moreso as the % retained grows in scenarios 6b and 6c. Minor decrease in shares going to manufacturers, wholesalers, and pharmacies (decreases grow larger as the % retained by PBMs increases). Minor changes in shares going to health plans.
**Scenario 6b:** PBMs Retain 10% of Insulin Rebates	45.08	-34%	73%	226%	118%	-24%	
**Scenario 6c:** PBMs Retain 12.5% of Insulin Rebates	43.95	-34%	71%	222%	115%	-23%	
**Scenario 7a:** Increase health plans' gross margin by 10%	46.24	-33%	76%	230%	155%	-24%	Share retained by health plans increases (decreases) as insurance margins increase (decrease). Shares of other participants decrease (increase) slightly.
**Scenario 7b:** Decrease health plans' gross margin by 10%	47.23	-33%	74%	228%	154%	-25%	
**Scenario 8:** Exclude biosimilar insulin glargine injection (Basaglar) from analysis	46.38	-33%	79%	229%	159%	-26%	In 2017-18, wholesalers and PBMs make slightly more when the biosimilar is excluded, suggesting that the biosimilar is slightly less profitable than average insulin. Minimal effect on other participants.

© 2021 Van Nuys K et al. *JAMA Health Forum.*

eReferences

1. CMS. 2014 Part D Rebate Summary for All Brand Name Drugs. U.S. Centers for Medicare & Medicaid Services. https://www.cms.gov/Research-Statistics-Data-and-Systems/Statistics-Trends-and-Reports/Information-on-Prescription-Drugs/PartD_Rebates#1-note. Published 2014. Accessed November 2, 2020.
2. Fein AJ. The Gross-to-Net Bubble Reached a Record $166 Billion in 2018. In: Institute DC, ed. Vol 20202019.
3. Fein AJ. *The 2020 Economic Report on U.S. Pharmacies and Pharmacy Benefit Managers.* Drug Channels Institute;2020.
4. MACPAC. Medicaid Drug Spending Trends Fact Sheet. In: Commission MaCPaA, ed. Washington, D.C.2019.

© 2021 Van Nuys K et al. *JAMA Health Forum.*
